# Processing-Driven Changes in Phenolic Composition and Antioxidant Capacity During Plum Wine Production from the ‘Stanley’ Cultivar

**DOI:** 10.3390/foods15081360

**Published:** 2026-04-14

**Authors:** Violeta Jevtovic, Khulood Fahad Saud Alabbosh, Buthainah Ameen Al Shankiti, Tarfah Abdulrahman M. Alaskar, Reem Ali Alyami, Vesna Stankov Jovanović, Jelena Nikolić, Pavle Mašković, Milan Mitić

**Affiliations:** 1 Chemistry Department, College of Science, University of Ha’il, Ha’il 81451, Saudi Arabia; v.jevtovic@uoh.edu.sa (V.J.); b.alshanqiti@uoh.edu.sa (B.A.A.S.); t.alaskar@uoh.edu.sa (T.A.M.A.); reem-alyami1@hotmail.com (R.A.A.); 2 Biology Department, College of Science, University of Ha’il, Ha’il 81451, Saudi Arabia; k.alabosh@uoh.edu.sa; 3 Department of Chemistry, Faculty of Science and Mathematics, University of Niš, 18000 Niš, Serbia; vesna.stankov-jovanovic@pmf.edu.rs (V.S.J.); jelena.cvetkovic@pmf.edu.rs (J.N.); 4 Department of Chemical Engineering, Faculty of Agronomy, University of Kragujevac, Cara Dušana 34, 32102 Cacak, Serbia; pavlem@kg.ac.rs

**Keywords:** phenolic profile, fermented fruit beverages, maceration, antioxidant activity, HPLC–DAD analysis, food processing, plum wine

## Abstract

Plum fruits are a valuable raw material to produce fermented beverages and a source of phenolic compounds with antioxidant properties. However, information on changes in phenolic composition during plum wine production is still limited. In this study, the evolution of phenolic compounds and antioxidant capacity during maceration and fermentation of wine from the ‘Stanley’ cultivar was investigated. Total phenolics, flavonoids, anthocyanins, and antioxidant capacity were determined spectrophotometrically, while individual compounds were identified by HPLC–DAD analysis. Eleven phenolic compounds were detected, including anthocyanins, hydroxycinnamic acids, and flavonols. Neochlorogenic acid, cyanidin-3-rutinoside, and rutin were the predominant compounds in fruits and wines. Phenolic content in plum skin was more than twofold higher than in whole fruit (445.20 vs. 198.32 mg GAE/100 g FW), with markedly higher anthocyanins (180.08 vs. 36.73 mg CGE/100 g FW), while juice showed much lower levels (89.32 mg GAE/L and 1.08 mg CGE/L). Maceration increased phenolic content and antioxidant activity, whereas fermentation led to a gradual decrease in most compounds, likely due to polymerization and degradation reactions. The wine produced contained 10.80 ± 0.15% (*v*/*v*) ethanol. Principal component analysis differentiated samples according to phenolic profile and fermentation stage.

## 1. Introduction

Plum (*Prunus salicina* L.), belonging to the Rosaceae family, is widely cultivated and consumed in many parts of the world, particularly in America, China, and Europe [1]. Plum fruits are valued for their attractive appearance, pleasant flavor, and significant content of biologically active constituents [2,3,4]. Because fully ripe plums are highly susceptible to postharvest deterioration, they are commonly processed into juices, jams, and canned products to extend their availability and reduce storage losses [5]. In parallel with growing consumer interest in beverages with distinctive sensory and functional characteristics, non-grape fruit wines such as blueberry, mango, mulberry, and plum wines have attracted increasing scientific and technological attention [6,7,8,9]. Fruit wine production is therefore recognized as an important processing route for horticultural raw materials, offering an opportunity to obtain value-added products from fruits with limited shelf life [10]. Phenolic compounds are important contributors to the antioxidant potential, color characteristics, and overall quality of fruit-derived beverages, while their composition may change considerably during technological processing and storage, directly affecting the properties of the final product [11]. These compounds include several groups, such as hydroxycinnamic acids, flavonols, and anthocyanins, which differ in their stability and reactivity during processing. Their transformation during maceration and fermentation may involve extraction, degradation, oxidation, and polymerization processes, ultimately influencing both the functional and sensory properties of the final product. Despite this, plum wine remains insufficiently studied, particularly regarding changes in bioactive composition during processing.

In Serbia, plum cultivation has a long agricultural and cultural tradition, and *Prunus domestica* L. remains the dominant species due to favorable climatic conditions, wide adaptability, and the nutritional and technological value of its fruits [2]. Serbia is among the leading plum-producing countries worldwide and ranks immediately after China and Romania, contributing approximately 4.1% of global production [12]. A substantial proportion of the harvest is used to produce the traditional distilled beverage ‘Šljivovica’, whereas fresh consumption remains comparatively limited [13]. Among the cultivars grown in Serbia, ‘Stanley’ is one of the most widely cultivated and technologically important cultivars, recognized as suitable for both fresh consumption and industrial processing, including fermented products [2,14].

Fruit cultivar not only influences the physicochemical characteristics of fresh fruits but also significantly affects the compositional quality of the resulting fruit wine. For this reason, the selection of raw material represents an important factor in fruit wine production. Previous studies [9,15] have reported the quality characteristics of plum wines produced from several Serbian cultivars; however, data on wine produced from the ‘Stanley’ cultivar remain limited.

Although plum wine is a promising value-added product derived from widely available horticultural raw material, its compositional changes and functional properties during processing remain insufficiently documented in the scientific literature. A clearer understanding of phenolic transformations during maceration and fermentation may contribute to better utilization of plum fruits and improved preservation of bioactive compounds in the final product. Therefore, the aim of this study was to evaluate the changes in phenolic composition, color parameters, and antiradical activity during the production of plum wine from the ‘Stanley’ cultivar commonly grown in Serbia.

## 2. Materials and Methods

### 2.1. Plant Material

Fruits of the ‘Stanley’ plum cultivar were harvested in September 2024 from a rural area with no known industrial or agricultural contamination sources in Blace, southeastern Serbia. The collected fruits were divided into separate portions for preparing peel, juice, and whole-fruit samples. Fruits were washed and manually pitted prior to further processing. A portion of the fresh fruits was manually peeled using a knife to obtain skin samples. Another portion was homogenized using a laboratory blender, and the juice was obtained by pressing the homogenized mass. Whole-fruit samples refer to homogenized fruits including both peel and pulp. The samples were individually frozen and stored at −20 °C until analysis. Extractions were then performed separately for whole fruits, peels, and juice to assess the distribution of bioactive compounds within different fruit tissues. The remaining fruits were used for the maceration and fermentation experiments.

### 2.2. Preparation of the Extracts

Frozen whole fruits, plum skins, and juice samples were thawed at room temperature and homogenized separately using a laboratory blender. Approximately 15 g of each homogenized sample (fruit mash, juice, or skin) was weighed into an Erlenmeyer flask and extracted with 20 mL of methanol. Ultrasonic extraction was performed twice in a standard laboratory ultrasonic bath (35 kHz, 120 W) for 20 min at room temperature. After each extraction step, the extracts were filtered, the filtrates were combined, and the final volume was adjusted to 50 mL with methanol. The prepared extracts were stored under refrigerated conditions until analysis. All analyses were performed in triplicate.

### 2.3. Plum Juice Preparation and Plum Wine Fermentation

Plum fruits were washed, pitted, and ground using a laboratory blender. The obtained must was treated with potassium metabisulphite (50 mg SO_2_/kg must) to prevent oxidation. Maceration was performed at 20 °C, and samples were collected at 24 h intervals throughout the process to monitor the selected parameters. After three days of maceration, the juice was separated from the pomace.

The juice obtained after maceration was diluted with distilled water in a 1:1 (*v*/*v*) ratio. The soluble solids content of the diluted juice was determined using a refractometer and was approximately 10–12 °Brix reflecting natural variability in the raw material. To adjust the sugar concentration to 21 °Brix, food-grade sucrose was added in calculated amounts based on mass balance, considering the initial soluble solids content and the total volume of the must. The mixture was thoroughly homogenized prior to fermentation, and the final °Brix value was verified by repeated refractometric measurements. This adjustment ensured suitable sugar content for alcoholic fermentation, as plum juice naturally contains insufficient fermentable sugars. Fermentation was carried out at 20 °C and pH 3.5 using 0.25 g/kg of commercial wine yeast *Saccharomyces cerevisiae* Spiriferm (Erbslöh, Geisenheim, Germany). The fermentation process continued until the residual reducing sugar content reached 4 g/L, achieved after six days. Samples were collected every 24 h during fermentation to follow changes in process parameters.

### 2.4. Physicochemical Characterization of Wine Samples

The pH values were determined using a microprocessor-based pH/mV/°C meter pH 212 (Hanna Instruments, Woonsocket, RI, USA). Total soluble solids (TSS) in the must were measured with a PAL-87S refractometer (Atago, Tokyo, Japan), and the results were expressed as °Brix. Alcohol content was determined after distillation of wine samples using a DMA 35 density meter (Anton Paar, Graz, Austria).

### 2.5. Determination of Total Phenolic Content (TPC)

Total phenolic content (TPC) was determined using the Folin–Ciocalteu reagent according to the method described by Negro et al. [15]. The results were expressed as mg gallic acid equivalents (GAE) per 100 g fresh weight (FW) of fruit or peel, or per liter of juice and wine.

### 2.6. Determination of Total Flavonoid Content (TFC)

Total flavonoid content (TFC) was measured by the colorimetric AlCl_3_ method described by Negro et al. [16]. The results were expressed as mg catechin equivalents (CE) per 100 g FW of fruit or peel, or per liter of juice and wine.

### 2.7. Determination of Total Anthocyanin Content (TAC)

Total anthocyanin content (TAC) was determined spectrophotometrically at 520 nm based on anthocyanin discoloration after the addition of SO_2_ (in the form of K_2_S_2_O_5_) at pH 1, according to Ribéreau-Gayon and Stonestreet [17].

### 2.8. Determination of Polymeric Color

Polymeric color in wine and must samples was determined spectrophotometrically according to the method described by Somers and Evans [18]. The method is based on the bleaching of monomeric anthocyanins after reaction with potassium metabisulphite, while polymerized anthocyanins remain unaffected and retain their color.

### 2.9. Quantitative Determination of Individual Phenolic Compounds by HPLC–DAD Analysis

Individual phenolic compounds were identified and quantified using an Agilent 1200 Series HPLC–DAD (Agilent Technologies, Santa Clara, CA, USA) system with gradient elution, following the procedure previously described by Jevtovic et al. [19]. Analyses were carried out using an HPLC–DAD system equipped with an Eclipse XDB C18 column (4.6 × 150 mm, Agilent Technologies, Santa Clara, CA, USA ), maintained at 25 °C, with a 5 µL injection volume. The mobile phase consisted of solvent A (2% formic acid in water) and solvent B (80% acetonitrile, 2% formic acid), and separation was achieved using a gradient. Identification was performed by comparing retention times and UV spectra with those of authentic standards, while quantification was based on external calibration curves.

The calibration equations, correlation coefficients (R^2^), limits of detection (LOD), and limits of quantification (LOQ) are presented in Table 1.

For compounds lacking corresponding analytical standards, identification was based on their retention time and UV–Vis spectra, while quantification was performed using calibration curves of structurally related compounds: chlorogenic acid for neochlorogenic acid, *p*-coumaric acid for *p*-coumaroylquinic acid, rutin for isorhamnetin glycoside, and cyanidin-3-glucoside for cyanidin-3-rutinoside. The results were expressed as mg/100 g FW or mg/L.

### 2.10. Determination of Antioxidant Capacity

The 1,1-diphenyl-2-picrylhydrazyl (DPPH) radical scavenging assay was performed according to the method described by Thaipong et al. [20]. The results were expressed as Trolox equivalent antioxidant capacity (mmol TE per 100 g FW or mmol TE/L).

ABTS (2,2′-azinobis 3-ethylbenzothiazoline-6-sulfonic acid) radical cation scavenging activity was determined according to the same procedure [20]. The results were also expressed as Trolox equivalent antioxidant capacity (mmol TE per 100 g FW or mmol TE/L).

### 2.11. Statistical Analysis

All analyses were performed in triplicate, and the results are presented as mean values ± standard error. Statistical differences among juice and wine samples were evaluated by one-way analysis of variance (ANOVA) followed by Tukey’s multiple comparison test at a significance level of *p* < 0.05.

Principal component analysis (PCA) was applied to examine relationships among wine samples collected at different stages of fermentation based on their phenolic composition. Prior to PCA, the data were auto-scaled to compensate for differences in the concentration ranges of individual phenolic compounds. Statistical analysis was performed using Statgraphics 18 software (Manugistics Inc., Rockville, MD, USA).

## 3. Results and Discussion

### 3.1. Chemical Composition of Different Parts of ‘Stanley’ Plum

To obtain an initial insight into the distribution of bioactive compounds within the raw material, total phenolic content, total flavonoid content, total anthocyanin content, antioxidant activity against DPPH and ABTS radicals, and the phenolic profile were determined separately in whole fruits, skins, and juice, as described in Section 2.2. This initial characterization enabled the identification of fruit tissues with the highest concentrations of key bioactive constituents. The results obtained are presented in Table 2.

The whole fruit of the ‘Stanley’ plum contained total phenolics of 198.32 ± 2.32 mg GAE/100 g FW, total flavonoids of 102.68 ± 1.15 mg CE/100 g FW, and total anthocyanins of 36.73 ± 0.98 mg CGE/100 g FW. A markedly higher concentration of all analyzed groups was observed in the peel, where TPC reached 445.20 ± 2.02 mg GAE/100 g FW, TFC 212.68 ± 1.80 mg CE/100 g FW, and TAC 180.08 ± 0.93 mg CGE/100 g FW. In contrast, juice contained substantially lower levels of these compounds, with values of 89.32 ± 1.02 mg GAE/L for TPC, 48.68 ± 0.96 mg CE/L for TFC, and 1.08 ± 0.13 mg CGE/L for TAC.

These results confirm that plum peel represents the principal reservoir of phenolic compounds and pigments in the fruit, which is consistent with previous reports for different plum cultivars [3,4,21,22]. Such distribution is particularly relevant for processing, since the transfer of peel-derived compounds during maceration directly determines the functional composition of the final wine. This pattern can be attributed to enhanced diffusion of phenolic compounds from the skin into the liquid phase during maceration.

The total phenolic content measured in ‘Stanley’ fruits in the present study was within the range previously reported for this cultivar. Kim et al. [23] reported 174.0 mg/100 g fresh weight, while Miletić et al. [24] observed pronounced year-to-year variation, with values ranging from 73 to 208 mg/100 g depending on harvest season and ripening stage. Such variability has been mainly associated with climatic factors, particularly temperature and precipitation during fruit development.

Compared with other plum species and cultivars, the obtained TPC values were lower than those reported for some *Prunus salicina* cultivars, where markedly higher concentrations have been described (Cevallos-Casals et al. [25]), but remained within the broader interval reported for commercial plum cultivars by other authors [26,27]. These differences confirm that phenolic accumulation in plums is influenced by both cultivar-specific characteristics and environmental conditions during growth.

The anthocyanin content observed in the present study was comparable to values previously reported for red-fleshed plum cultivars and hybrids, where concentrations between 33 and 173 mg/100 g have been reported (Cevallos-Casals et al. [25]), and consistent with the range reported by Wu et al. [28]. Lower anthocyanin levels were reported for some *Prunus domestica* cultivars by Franke et al. [29], indicating considerable variability among plum genotypes.

A similar distribution pattern between fruit tissues was reported by Trendafilova et al. [4], who showed that anthocyanin concentrations in ‘Čačanska lepotica’ were markedly higher in the peel than in the whole fruit, confirming that skin tissue represents the main site of anthocyanin accumulation in plum fruits.

Antioxidant capacity, evaluated by DPPH and ABTS assays, followed the same distribution pattern observed for phenolic compounds. Peel extracts exhibited the highest DPPH radical scavenging activity (4.52 ± 0.28 mmol TE/100 g FW), followed by whole-fruit extracts (2.03 ± 0.23 mmol TE/100 g FW), whereas juice showed the lowest activity (0.90 ± 0.12 mmol TE/L) (Table 2). A similar trend was observed for ABTS values, with peel extracts showing approximately twofold higher antioxidant capacity than whole-fruit extracts.

The stronger antioxidant response of peel extracts is consistent with their higher phenolic and anthocyanin concentrations, indicating that these compounds are the main contributors to radical scavenging activity in plum tissues. Similar relationships between phenolic accumulation and antioxidant capacity have been reported for different *Prunus domestica* cultivars [22,30,31,32]. From a processing perspective, these results further emphasize the importance of preserving peel contact during maceration to maximize the transfer of antioxidant-active compounds into the wine matrix.

#### Individual Phenolic Compounds

**Figure 1:**
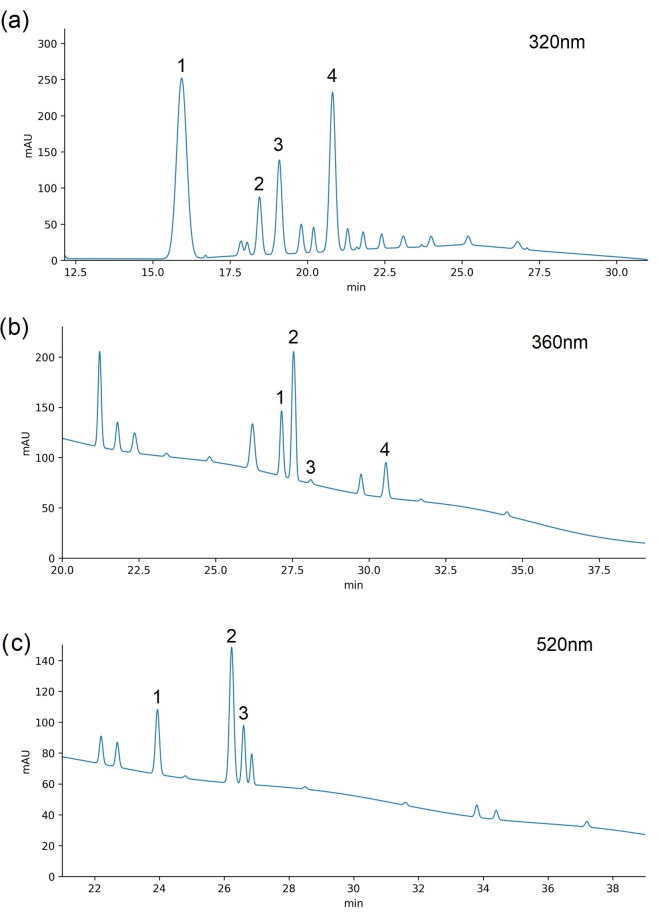
HPLC chromatograms of methanolic plum extracts with identified phenolic compounds: (**a**) hydroxycinnamic acids at 320 nm—1: neochlorogenic acid; 2: caffeic acid; 3: chlorogenic acid; 4: *p*-coumaroylquinic acid; (**b**) flavonols at 360 nm—1: hyperoside; 2: rutin; 3: isoquercetin; 4: isorhamnetin glycoside; (**c**) anthocyanins at 520 nm—1: cyanidin-3-glucoside; 2: cyanidin-3-rutinoside; 3: peonidin-glucoside.

A total of eleven individual phenolic compounds were identified based on their chromatographic retention characteristics and UV–Vis spectral profiles and classified into three main groups: hydroxycinnamic acids (neochlorogenic acid, caffeic acid, chlorogenic acid, and 3-*p*-coumaroylquinic acid), flavonols (quercetin-rutinoside, quercetin-3-glucoside, quercetin-3-galactoside, and isorhamnetin-glycoside), and anthocyanins (cyanidin-3-glucoside, cyanidin-3-rutinoside, and peonidin-3-glucoside) (Table 2, Figure 1).

The identified profile confirms that hydroxycinnamic acids represent the dominant non-pigment phenolic fraction, whereas flavonols and anthocyanins were predominantly concentrated in the peel, where their levels were approximately two- to threefold higher than in the whole fruit.

The dominant individual phenolic compounds identified in both ‘Stanley’ plum fruit and juice were hydroxycinnamic acid derivatives, with neochlorogenic acid as the prevailing component, reaching 18.38 mg/100 g fresh weight in the fruit and 25.32 mg/L in the juice, whereas chlorogenic acid was present at lower concentrations (3.22 mg/100 g fresh weight and 4.16 mg/L, respectively). In the juice, hydroxycinnamic acids accounted for more than 95% of the identified individual phenolics and represented 41.22% of total phenolic content, confirming their dominant contribution to the phenolic profile, in agreement with previous findings [33]. This predominance was calculated from the sum of identified hydroxycinnamic acids relative to the total concentration of quantified individual phenolic compounds in juice, indicating that neochlorogenic, chlorogenic, caffeic, and *p*-coumaroylquinic acids together constituted a major part of the identified phenolic fraction. Higher concentrations of neochlorogenic acid were reported for cultivars such as ‘Jodžo’ and ‘Čačanska najbolja’, where values reached 50.3–55.4 and 39.8–55.5 mg/100 g fresh weight, respectively [34]. The values obtained in the present study remained within the broader range reported by Jaiswal et al. [35], who found concentrations ranging from trace amounts to 52.2 mg/100 g fresh weight, depending on cultivar. A similar predominance of neochlorogenic over chlorogenic acid was also observed in ‘Čačanska lepotica’ [4], while Usenik et al. [36] likewise identified neochlorogenic acid as the major hydroxycinnamic acid derivative during plum ripening.

Anthocyanins represented the second most abundant group of identified phenolics in whole fruit and the dominant group in peel, accounting for 8.9% and 13.2% of total phenolic content, respectively (Table 2). Among them, cyanidin-3-O-rutinoside was the predominant anthocyanin, reaching 40.22 mg/100 g fresh weight in peel and 13.00 mg/100 g fresh weight in fruit. The concentrations of cyanidin-3-O-glucoside and peonidin-3-O-glucoside were considerably lower, amounting to 14.36 and 4.18 mg/100 g fresh weight in peel, and 2.82 and 1.81 mg/100 g fresh weight in fruit, respectively. Cyanidin-3-O-rutinoside and cyanidin-3-O-glucoside are recognized as characteristic anthocyanins in plum fruits and have been reported as major pigments in numerous cultivars [37]. A similar anthocyanin profile, with cyanidin-3-glucoside, cyanidin-3-rutinoside, and peonidin-3-glucoside as dominant components, was also described by Walkowiak-Tomczak [38].

Flavonols were less abundant than hydroxycinnamic acids and anthocyanins, accounting for 6.10% of total phenolic content in whole fruit and 8.37% in peel. Rutin was the predominant flavonol in both tissues, followed by isoquercetin and isorhamnetin derivatives, whereas hyperoside was present at the lowest concentration. The predominance of rutin agrees with previous reports describing it as one of the principal flavonols in plum fruits [39]. A similar flavonol pattern, with rutin, hyperoside, and isoquercetin as dominant compounds, was reported for the Slovenian cultivar ‘Domača češplja’, although additional flavonol glycosides such as kaempferol and isorhamnetin derivatives were also identified [40]. Treutter et al. [41] likewise detected isorhamnetin glycosides in plum fruit, while quantitative data for these compounds remain limited in the available literature.

### 3.2. Extraction Process

Since the highest concentrations of target compounds were identified in the fruit skin, the processing protocol included a maceration stage in which the solid parts remained in contact with the juice to promote their transfer into the liquid phase. Maceration of crushed fruit is a well-established step in fruit wine production because it enhances both color development and aromatic complexity. Prolonged contact between the juice and skin facilitates the release of phenolic substances, anthocyanins, and volatile constituents that contribute to the overall quality of the final product. This process is primarily governed by diffusion mechanisms and the progressive disruption of plant cell walls, which enhances the release of intracellular compounds into the surrounding liquid phase.

The duration of maceration is considered one of the key factors affecting extraction efficiency, particularly for phenolic compounds in the must. Previous studies have shown that the most favorable color characteristics and anthocyanin concentrations are generally achieved when maceration lasts between three and six days, while extended skin contact may lead to a gradual decline in color intensity [42].

For this reason, pomace from the ‘Stanley’ plum variety was macerated at 20 °C for three days. The evolution of individual phenolic compounds during this period was subsequently monitored. Table 3 presents the changes observed in hydroxycinnamic acids, flavonols, and anthocyanins determined by HPLC analysis.

The concentrations of phenolic compounds changed throughout maceration, although the extent of variation differed among compound groups. In general, most compounds showed a noticeable increase between day 0 and day 2, whereas the changes recorded on day 3 were less pronounced or not statistically significant. Skin contact during maceration promoted the transfer of phenolic constituents into the juice, resulting in higher concentrations and the detection of more compounds. This increase is primarily driven by the progressive breakdown of cell structures and improved mass transfer between the solid and liquid phases during maceration. Hydroxycinnamic acids are typically more readily extracted due to their localization in vacuoles and cell walls, whereas anthocyanins, being predominantly located in the skin, require sufficient contact time for efficient release.

A comparison between plum juice and must after three days of maceration (M3) clearly demonstrates the strong effect of skin contact on phenolic extraction. Anthocyanin concentration in plum juice remained very low (1.08 mg/L, Table 2), whereas after maceration it increased markedly to 78.23 mg/L in M3 (Table 3). A similar pattern was observed for total phenolic content, which increased from 89.32 mg GAE/L in plum juice to 1605.22 mg GAE/L after maceration.

Since total phenolics are closely associated with antioxidant capacity and potential biological activity, the substantially higher phenolic concentration in M3 was accompanied by a pronounced increase in antioxidant activity. In contrast, plum juice without maceration showed considerably lower antioxidant potential. This relationship reflects not only the increased concentration of phenolic compounds but also their structural characteristics, which influence their radical-scavenging capacity. The results confirm that short-term maceration plays a decisive role in the release of skin-associated phenolic constituents, especially anthocyanins, which are predominantly concentrated in the outer fruit tissues.

### 3.3. Fermentation Process

Fermentation represents a key stage in fruit wine production, during which sugars are converted into ethanol, and a wide range of secondary metabolites, such as esters and higher alcohols, are formed, contributing significantly to the final quality of the wine. Previous studies have shown that several factors influence the physicochemical and sensory properties of fruit wines, including yeast strain selection [5], mixed-culture inoculation strategies and inoculum ratios [43], and fermentation conditions such as temperature and pH [44].

Alcohol content is one of the main indicators used to monitor fermentation efficiency and assess fruit wine quality. As shown in Table 4, ethanol concentration increased progressively from an initial value of 0%, reflecting active sugar conversion by yeast metabolism. After 144 h of fermentation, ethanol content reached 10.70%, indicating efficient alcoholic fermentation and satisfactory sugar utilization [45].

During the early fermentation stage, ethanol formation proceeded rapidly due to intensive sugar metabolism and high yeast activity. As fermentation progressed, the rate of alcohol production gradually declined, mainly because of substrate depletion, increasing ethanol concentration, and less favorable environmental conditions for yeast growth. After approximately five days, ethanol synthesis slowed down and eventually stabilized in the final stage of fermentation.

Table 4 also summarizes the evolution of total phenolic content (TPC), total flavonoid content (TFC), total anthocyanin content (TAC), percentage of polymeric color, antioxidant activity determined by spectrophotometric methods, and the phenolic profile obtained by HPLC-DAD analysis in the initial must and plum wines.

**Figure 2:**
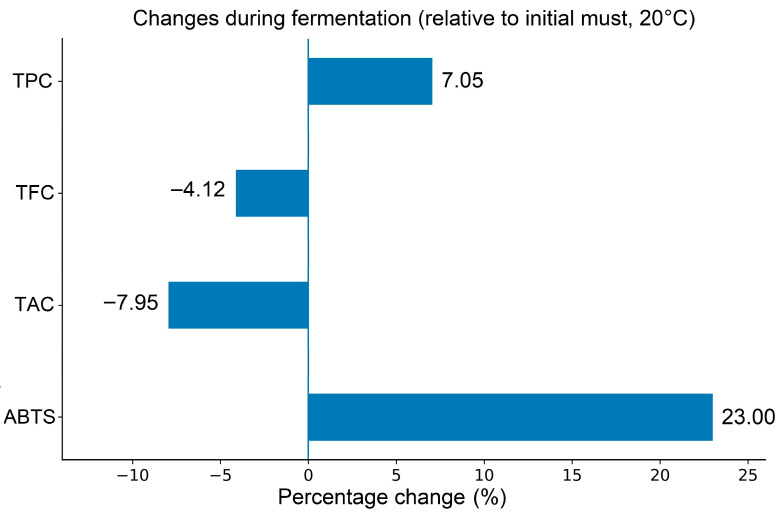
Percentage changes in total phenolic content (TPC), total flavonoid content (TFC), total anthocyanin content (TAC), and ABTS antioxidant activity relative to the initial must during fermentation at 20 °C (*n* = 3).

The evolution of total phenolic content during fermentation showed moderate fluctuations rather than a uniform trend. An initial increase was observed from day 0 to day 2, followed by a temporary decrease on day 3, while a gradual rise was again recorded from day 4 to day 6. The initial must (W0) contained 802.62 mg GAE/L of total phenolics, whereas the highest value was measured at the end of fermentation, reaching 859.22 mg GAE/L, corresponding to an overall increase of 7.05% (Figure 2). In contrast, total flavonoid content decreased slightly during the same period, with a final reduction of 4.12% (Figure 2).

Changes in total phenolic content during fruit wine fermentation have been reported inconsistently in the literature. Fluctuating phenolic levels were described during kiwi wine fermentation by Qi et al. [46], whereas a slight decrease in total phenolics was observed in pomegranate wine [47]. These differences are generally attributed to the characteristics of the fruit matrix, extraction dynamics, and fermentation conditions, all of which influence phenolic stability and transformation [48]. Such behavior confirms that fermentation is not only a biochemical conversion of sugars into ethanol, but also a dynamic stage in which phenolic compounds undergo continuous extraction, transformation, and partial degradation, directly affecting the functional quality of the final wine. These processes are influenced by changes in ethanol concentration, pH, and yeast activity, which further affect phenolic stability and promote their transformation during fermentation.

In the initial must, total anthocyanin content (TAC) was 39.11 mg CGE/L. During the first days of fermentation, TAC remained relatively stable, after which a gradual decrease became evident. By day 6, TAC declined significantly to 36.00 mg CGE/L, representing a reduction of 7.95% (Figure 2). This decrease is mainly associated with the progressive polymerization of monomeric anthocyanins during fermentation. These reactions contribute to the formation of more stable pigment complexes, reducing the content of free anthocyanins.

Color polymerization data additionally confirmed this transformation: the percentage of polymeric color increased from 20.2% in the initial must to 50.8% at the end of fermentation (Table 4). Similar trends were reported by Bimpilas et al. [49], who observed comparable fluctuations in TPC, TFC, and TAC during fermentation of Merlot wine.

The antioxidant capacity of plum wine during fermentation, assessed using two different assays, showed distinct patterns of change over the six-day fermentation period (Table 4). For the DPPH assay, no statistically significant difference was observed between days 0 and 6, whereas the ABTS assay revealed a significant difference between the initial and final fermentation day (Figure 2). This divergence indicates that antioxidant assays based on different reaction mechanisms may respond differently to compositional changes occurring during fermentation.

A similar divergence between DPPH and ABTS responses has been reported by other authors. Thus, Akbulat et al. [50] found that DPPH values decreased during the fermentation of black carrot juice, while ABTS values increased. Such differences suggest that antioxidant evolution during fermentation depends not only on the total amount of phenolic compounds, but also on changes in their composition and reactivity. This observation also supports the strong relationship between total phenolic content and ABTS antioxidant activity, since each phenolic compound may exhibit different antioxidant potential depending on its chemical structure. On the other hand, Skroza et al. [51] reported that synergistic or antagonistic interactions among polyphenols may additionally influence the measured antioxidant response.

The changes in individual phenolic fractions were further evaluated during the six days of fermentation. Fermentation reduced the total concentration of identified individual phenolics by 6.68%, whereas TPC increased by 7.05% (Figure 2). This apparent discrepancy can be explained by the different analytical principles of the applied methods. While HPLC quantifies only selected identified phenolic compounds, the Folin–Ciocalteu assay reflects the sample’s overall reducing capacity and may also respond to other reducing substances and transformed phenolic derivatives formed during fermentation. The spectrophotometric assay measures not only phenolic compounds but also other reducing substances, such as ascorbic acid, which can react with the Folin–Ciocalteu reagent. As emphasized by Prior et al. [52], the TPC assay essentially reflects the reducing capacity of the sample and therefore behaves similarly to an antioxidant test. Consequently, TPC values should not be overinterpreted and must be considered with caution when compared with chromatographically quantified phenolics.

**Figure 3:**
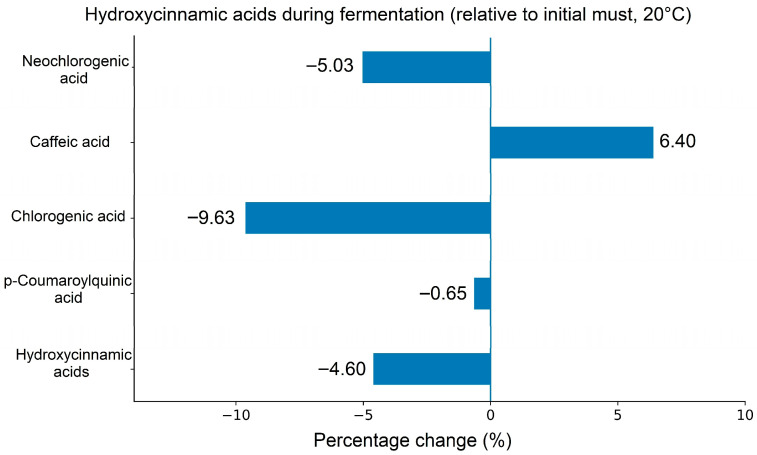
Percentage changes in individual hydroxycinnamic acids and their total content during fermentation at 20 °C (*n* = 3).

To further clarify the changes observed at the level of individual phenolic compounds, the behavior of hydroxycinnamic acids during fermentation was examined separately. Overall, fermentation caused a slight decrease in the total concentration of hydroxycinnamic acids, amounting to 4.60% (Figure 3). Hydroxycinnamic acids are recognized as important contributors to the phenolic profile and antioxidant-related properties of plant-derived matrices, although their abundance and behavior may vary depending on the raw material and processing conditions [53]. Among this group, neochlorogenic acid was the dominant compound in plum wine (Table 4), and its concentration decreased by 5.03% during fermentation. Among the minor hydroxycinnamic acids, *p*-coumaroylquinic acid was also present at a relatively high concentration and showed the smallest decrease (0.65%). In contrast, chlorogenic acid exhibited the greatest decline (9.63%), whereas caffeic acid increased by 6.40%.

These compound-specific changes are consistent with previous reports indicating that hydroxycinnamic acids may exhibit different trends during fermentation. Alencar et al. [54] reported a decrease in caffeic acid during fermentation, which is not consistent with the results obtained in this study. In contrast, Plavša et al. [55] observed a significant increase in hydroxybenzoic and hydroxycinnamic acids, including caffeic acid, in Teran red wine, which aligns with our findings. Similarly, Gayot et al. [56] found no substantial change in chlorogenic acid during apple juice fermentation, while caffeic acid increased and 4-*p*-coumaroylquinic acid remained stable. Ramos et al. [57] also reported a higher final concentration of this compound during grape wine fermentation at 14 °C.

Taken together, these findings suggest that the evolution of hydroxycinnamic acids during fermentation is governed by compound-dependent transformation pathways rather than by a single uniform trend, further highlighting the complexity of phenolic changes in plum wine.

In general, the hydroxycinnamic acid pattern observed in Stanley plum wine is consistent with literature data reported for other plum cultivars, although quantitative differences are expected due to genotype and processing conditions [54,55,56,57].

A similar trend was observed for anthocyanins. As shown in Table 4, three anthocyanins were identified in plum wine, including two cyanidin derivatives and one peonidin derivative. Cyanidin-3-*O*-rutinoside was the predominant anthocyanin, accounting for 70.81–73.51% of total anthocyanins, followed by cyanidin-3-*O*-glucoside (19.70–21.57%) and peonidin-3-*O*-glucoside (6.17–8.13%). This distribution indicates that cyanidin derivatives are the major anthocyanins in the plum wines produced, which is consistent with the typical anthocyanin profile of plum fruit.

**Figure 4:**
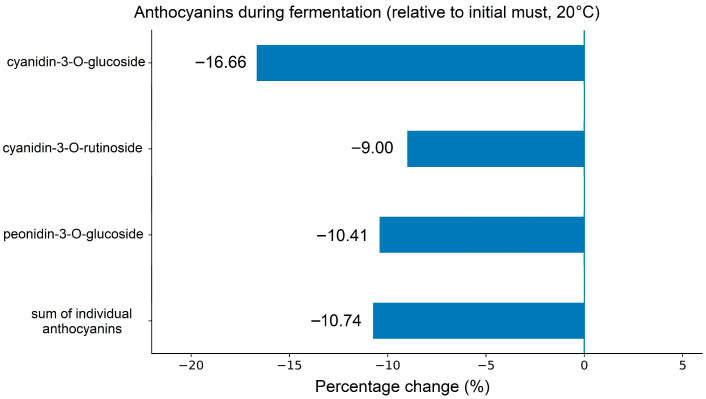
Percentage changes in individual anthocyanins and their total content relative to the initial must during fermentation at 20 °C (*n* = 3).

As illustrated in Figure 4, fermentation decreased in all identified anthocyanins relative to their initial concentrations in the must. The concentration of cyanidin-3-*O*-glucoside decreased by 16.66%, cyanidin-3-*O*-rutinoside by 9.00%, and peonidin-3-*O*-glucoside by 10.41%, while the sum of individual anthocyanins declined by 10.74%. These changes are consistent with previous findings indicating that anthocyanins may be partially degraded, adsorbed by yeast cells, or transformed into more stable derivative forms during alcoholic fermentation [58]. Such transformations are particularly important from a technological perspective, since they contribute to the progressive shift from monomeric anthocyanins toward more stable pigments that support long-term wine color stability [1].

**Figure 5:**
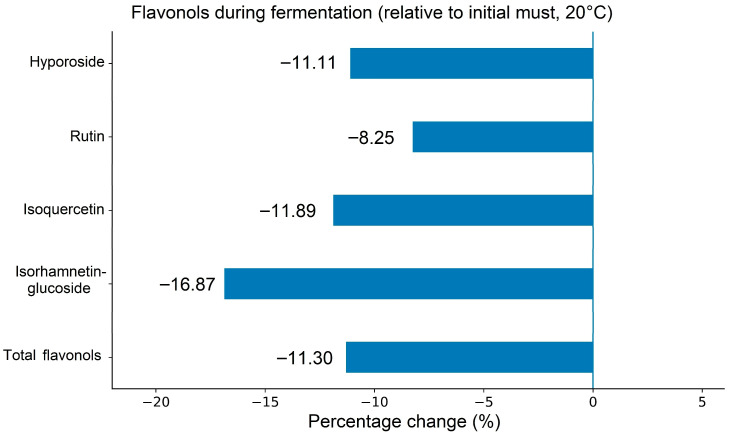
Percentage changes in individual flavonols and their total content (Flav.) relative to the initial must during fermentation at 20 °C (*n* = 3).

Other phenolic constituents detected in the initial must and plum wines belonged to the flavonol group. Table 4 shows that four flavonols were identified and quantified, among which rutin was the dominant compound in all samples, in agreement with the known phenolic profile of plum fruit. During fermentation, rutin concentration decreased, indicating that flavonols were also affected by the transformations occurring throughout this stage. The percentage losses of individual flavonols are shown in Figure 5. Overall, these results confirm that fermentation not only modifies the total phenolic balance of plum wine, but also reshapes the distribution of individual phenolic groups, which may ultimately influence its antioxidant and sensory properties. Taken together, these findings highlight that fermentation represents a critical stage of phenolic transformation, where carefully controlled conditions can be used to optimize phenolic composition, enhance color stability, and improve the overall functional quality of plum wine.

### 3.4. PCA—The Relationship Between Phenolic Compounds and Fermentation Process Time

**Figure 6:**
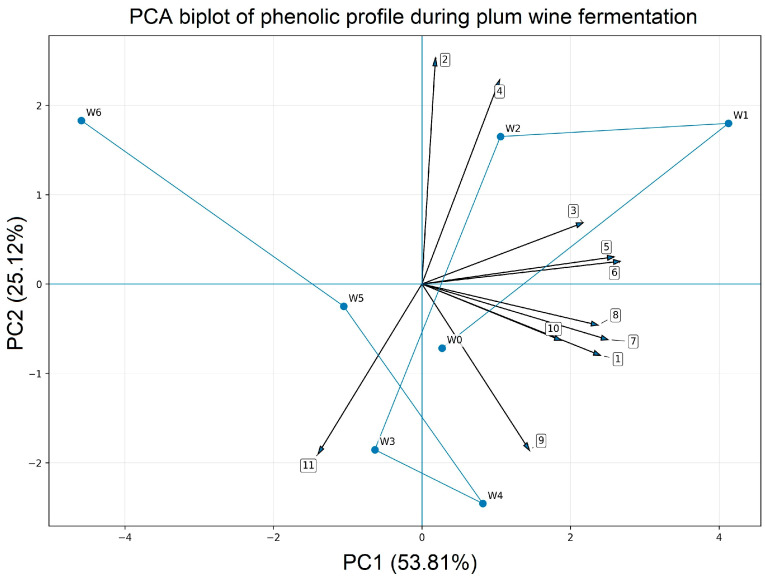
Principal component analysis (PCA) biplot of the phenolic profile of plum wine samples collected during fermentation. W0 represents the initial must, while W1–W6 correspond to samples collected after 1–6 days of fermentation, respectively. Numbered vectors denote individual phenolic compounds-1: neochlorogenic acid; 2: caffeic acid; 3: chlorogenic acid; 4: *p*-coumaroylquinic acid; 5: hyperoside; 6: rutin; 7: isoquercetin; 8: isorhamnetin-glycoside; 9: cyanidin-glucoside; 10: cyanidin-rutinoside; 11 peonidin-glucoside.

To better evaluate the relationship between fermentation time and changes in individual phenolic compounds, principal component analysis (PCA) was applied to the phenolic dataset. The PCA biplot (Figure 6) showed a clear distribution of samples by fermentation stage, indicating progressive changes in the phenolic profile throughout the process. The first two principal components explained 78.93% of the total variance, with PC1 and PC2 accounting for 53.81% and 25.12%, respectively. The positioning of the samples and the direction of the variable vectors indicate that fermentation time influenced the relative contribution of individual phenolic compounds, confirming that the phenolic profile evolved continuously throughout the six-day process.

## 4. Conclusions

This study provides new insight into the phenolic composition and antioxidant potential of wine produced from the Stanley plum variety, an important cultivar in Serbia and the Balkan region. The results showed that maceration significantly enhanced the extraction of phenolic compounds from plum skin, resulting in higher levels of total phenolics, anthocyanins, flavonoids, flavanols, and hydroxycinnamic acids in the must. During fermentation, the phenolic profile continued to change, with a slight increase in total phenolic content and a decrease in anthocyanins, while individual phenolic groups showed compound-specific behavior. Overall, the Stanley plum variety proved to be a suitable raw material for plum wine production, yielding a product with considerable antioxidant activity and a rich phenolic profile. The results contributed to a better understanding of the compositional changes occurring during this process.

The final plum wine (W6) was characterized by the highest total phenolic content (859.22 mg/L), accompanied by an increase in antioxidant activity (ABTS: 4.92; DPPH: 3.02), while polymeric color reached 50.8%, indicating progressive phenolic transformations during fermentation.

Further research should focus on different plum cultivars, fermentation conditions, and storage stability to better understand phenolic transformations and optimize the quality of plum wine.

## Figures and Tables

**Table 1:** Validation parameters for eight phenolic compounds used for HPLC-DAD analysis.

Compound	Calibration Curve	(R^2^)	LOD ^1^ (μg/mL)	LOQ ^2^ (μg/mL)
Caffeic acid	y=32,505.2x+0.3	0.9997	0.32	1.36
Chlorogenic acid	y=20,325.2x−0.2	0.9998	0.35	1.16
*p*-Coumaric acid	y=39,215.2x−1.0	0.9992	0.55	1.67
Hyperoside	y=5554.0x+0.5	0.9996	0.30	1.00
Rutin	y=4362.2x−0.5	0.9998	0.33	1.10
Isoquercetin	y=4988.1x+0.3	0.9996	0.48	1.45
Cyanidin-3-glucoside	y=5726.8x+0.2	0.9997	0.30	1.00
Peonidin-3-glucoside	y=4086.0x+0.5	0.9998	0.29	0.97

^1^ LOD-limit of detection; ^2^ LOQ-limit of determination.

**Table 2:** Total phenols (TPC), total flavonoids (TFC), and total anthocyanins (TAC) content and individual phenolic composition of the fruit, fruit skin and fruit juice of the ‘Stanley’ cultivar.

	Fruit (mg/100 g FW)	Skin (mg/100 g FW)	Juice (mg/L)
TPC (mg GAE/L)	198.32 ± 2.32 ^b^	445.20 ± 2.02 ^a^	89.32 ± 1.02 ^c^
TFC (mg CE/L)	102.68 ± 1.15 ^b^	212.68 ± 1.80 ^a^	48.68 ± 0.96 ^c^
TAC (mg CGE/L)	36.73 ± 0.98 ^b^	180.08 ± 0.93 ^a^	1.08 ± 0.13 ^c^
DPPH (mmol TE/L)	2.03 ± 0.23 ^b^	4.52 ± 0.22 ^a^	0.90 ± 0.12 ^c^
ABTS (mmol TE/L)	2.56 ± 0.11 ^b^	5.38 ± 0.30 ^a^	1.02 ± 0.13 ^c^
Hydroxycinnamic acids			
Neochlorogenic acid	18.38 ± 0.23 ^c^	23.32 ± 0.20 ^b^	25.32 ± 0.28 ^a^
Caffeic acid	2.38 ± 0.09 ^c^	4.32 ± 0.12 ^a^	2.52 ± 0.12 ^b^
Chlorogenic acid	3.22 ± 0.12 ^c^	6.52 ± 0.22 ^a^	4.16 ± 0.12 ^b^
*p*-Coumaroylquinic acid	3.81 ± 0.10 ^c^	5.32 ± 0.25 ^a^	4.82 ± 0.20 ^b^
Σ _Hydroxycinnamic acids_	27.79	39.48	36.82
Flavonols			
Hyperoside	1.38 ± 0.08 ^b^	6.78 ± 0.11 ^a^	0.23 ± 0.02 ^c^
Rutin	5.82 ± 0.19 ^b^	20.20 ± 0.22 ^a^	0.82 ± 0.04 ^c^
Isoquercetin	2.52 ± 0.12 ^b^	5.28 ± 0.16 ^a^	0.67 ± 0.02 ^c^
Isorhamnetin-glycoside	2.38 ± 0.12 ^b^	5.02 ± 0.12 ^a^	0.48 ± 0.03 ^c^
Σ_Flavonols_	12.10	*37.28*	2.20
Anthocyanins			
Cyanidin-glucoside	2.82 ± 0.02 ^b^	14.36 ± 0.28 ^a^	0.09 ± 0.01 ^c^
Cyanidin-rutinoside	13.00 ± 0.10 ^b^	40.22 ± 0.30 ^a^	0.23 ± 0.02 ^c^
Peonidin-3-glucoside	1.81 ± 0.01 ^b^	4.18 ± 0.10 ^a^	0.03 ± 0.00 ^c^
Σ _Anthocyanins_	17.63	58.76	0.35
Σ _Phenolic Compounds_	57.52	135.52	39.37

Results for whole fruit and fruit skin are expressed as mg/100 g fresh weight (FW), whereas results for juice are expressed as mg/L. TPC is expressed as mg gallic acid equivalents (GAE), TFC as mg catechin equivalents (CE), TAC as mg cyanidin-3-glucoside equivalents (CGE), and antioxidant activity as mmol Trolox equivalents (TE). Different letters indicate statistically significant (*p* < 0.05) differences between the quantitative values of compounds.

**Table 3:** Total phenolic content (TPC), total flavonoid content (TFC), total anthocyanin content (TAC), as well as individual phenolic composition during maceration. All individual compounds and their summed values are expressed in mg/L.

	M1 (24 h)	M2 (48 h)	M3 (72 h)
TPC (mg GAE/L)	722.52 ± 2.82 ^c^	1321.12 ± 3.02 ^b^	1605.22 ± 3.82 ^a^
TFC (mg CE/L)	139.95 ± 1.95 ^c^	298.50 ± 1.80 ^b^	322.12 ± 1.96 ^a^
TAC (mg CGE/L)	38.02 ± 0.98 ^c^	69.52 ± 1.53 ^b^	78.23 ± 2.14 ^a^
DPPH (mmol TE/L)	3.62 ± 0.43 ^b^	5.02 ± 0.42 ^a^	5.76 ± 0.52 ^a^
ABTS (mmol TE/L)	5.83 ± 0.51 ^c^	8.80 ± 0.50 ^b^	9.40 ± 0.44 ^a^
Polymeric color (%)	12.6	15.3	20.2
Hydroxycinnamic acids			
Neochlorogenic acid	67.24 ± 0.65 ^c^	90.70 ± 0.52 ^b^	91.38 ± 0.16 ^a^
Caffeic acid	6.96 ± 0.18 ^c^	10.76 ± 0.20 ^b^	11.24 ± 0.32 ^a^
Chlorogenic acid	17.36 ± 0.42 ^c^	29.84 ± 0.18 ^b^	30.10 ± 0.26 ^a^
*p*-Coumaroylquinic acid	14.52 ± 0.50 ^c^	21.02 ± 0.20 ^b^	21.48 ± 0.20 ^a^
Σ _Hydroxycinnamic acids_	106.08	152.32	154.40
Flavonols			
Hyperoside	1.44 ± 0.15 ^b^	2.44 ± 0.12 ^a^	2.70 ± 0.18 ^a^
Rutin	5.20 ± 0.22 ^c^	7.69 ± 0.14 ^b^	8.24 ± 0.40 ^a^
Isoquercetin	3.78 ± 0.20 ^c^	6.04 ± 0.10 ^b^	6.56 ± 0.28 ^a^
Isorhamnetin-glycoside	1.92 ± 0.08 ^c^	2.64 ± 0.10 ^b^	3.92 ± 0.14 ^a^
Σ Flavonols	12.34	19.08	21.48
Anthocyanins			
Cyanidin-glucoside	4.76 ± 0.24 ^c^	10.84 ± 0.24 ^b^	11.52 ± 0.28 ^a^
Cyanidin-rutinoside	16.10 ± 0.42 ^b^	38.10 ± 0.32 ^a^	38.62 ± 0.42 ^a^
Peonidin-3-glucoside	1.84 ± 0.16 ^c^	4.04 ± 0.10 ^b^	4.42 ± 0.18 ^a^
Σ _Anthocyanins_	21.70	42.14	54.56
Σ _Phenolic _Compounds	140.12	213.54	230.44

M1, M2, and M3 correspond to samples collected after 24, 48, and 72 h of maceration, respectively. TPC is expressed as mg gallic acid equivalents (GAE), TFC as mg catechin equivalents (CE), TAC as mg cyanidin-3-glucoside equivalents (CGE), and antioxidant activity as mmol Trolox equivalents (TE). Different letters indicate statistically significant (*p* < 0.05) differences between the quantitative values of compounds.

**Table 4:** Total phenolic content (TPC), total flavonoid content (TFC), total anthocyanin content (TAC), as well as individual phenolic composition during fermentation. All individual compounds and their summed values are expressed in mg/L.

	W0 (Must)	W1 (24 h)	W2 (48 h)	W3 (72 h)	W4 (96 h)	W5 (120 h)	W6 (144 h)
TPC (mg GAE/L)	802.62 ± 1.91 ^f^	803.65 ± 2.38 ^f^	820.55 ± 2.18 ^d^	810.56 ± 3.08 ^e^	838.72 ± 3.20 ^c^	845.66 ± 2.88 ^b^	859.22 ± 3.05 ^a^
TFC (mg CE/L)	155.05 ± 0.98 ^b^	158.54 ± 1.12 ^a^	152.34 ± 1.15 ^c^	146.58 ± 1.25 ^d^	148.33 ± 0.98 ^de^	148.84 ± 1.12 ^d^	148.66 ± 2.02 ^d^
TAC (mg CGE/L)	39.11 ± 1.07 ^a^	39.20 ± 1.35 ^a^	39.16 ± 1.30 ^a^	36.12 ± 1.23 ^c^	37.72 ± 1.06 ^b^	37.55 ± 1.52 ^b^	36.00 ± 1.18 ^c^
DPPH (mmol TE/L)	2.98 ± 0.26 ^a^	3.05 ± 0.33 ^a^	2.90 ± 0.48 ^a^	2.92 ± 0.58 ^a^	2.95 ± 0.76 ^a^	3.02 ± 0.57 ^a^	3.02 ± 0.78 ^a^
ABTS (mmol TE/L)	4.00 ± 0.22 ^b^	4.28 ± 0.38 ^b^	4.36 ± 0.55 ^ab^	4.55 ± 0.48 ^ab^	4.50 ± 0.60 ^ab^	4.60 ± 0.52 ^ab^	4.92 ± 0.60 ^a^
Polymeric color (%)	20.2 ± 0.1	28.2 ± 0.1	35.6 ± 0.1	36.7 ± 0.1	44.3 ± 0.0	46.8 ± 0.1	50.8 ± 0.1
Ethanol (%)	0.0 ± 0.0	4.0 ± 0.0	6.5 ± 0.1	8.7 ± 0.1	10.1 ± 0.0	10.7 ± 0.1	10.7 ± 0.1
Hydroxycinnamic acids							
Neochlorogenic acid	45.69 ± 0.38 ^c^	47.40 ± 0.30 ^a^	47.05 ± 0.32 ^a^	46.52 ± 0.28 ^b^	47.20 ± 0.38 ^a^	46.40 ± 0.20 ^b^	43.39 ± 0.26 ^d^
Caffeic acid	5.62 ± 0.18 ^c^	6.07 ± 0.15 ^a^	5.76 ± 0.10 ^bc^	4.92 ± 0.10 ^d^	4.63 ± 0.12 ^e^	5.05 ± 0.14 ^d^	5.98 ± 0.09 ^b^
Chlorogenic acid	15.05 ± 0.30 ^b^	15.52 ± 0.32 ^a^	14.25 ± 0.18 ^c^	13.80 ± 0.13 ^de^	14.05 ± 0.12 ^cd^	14.28 ± 0.10 ^c^	13.60 ± 0.09 ^e^
*p*-Coumaroylquinic acid	10.74 ± 0.20 ^b^	11.21 ± 0.30 ^a^	11.03 ± 0.18 ^ab^	10.49 ± 0.16 ^c^	10.10 ± 0.12 ^d^	10.78 ± 0.10 ^bc^	10.67 ± 0.12 ^c^
Σ Hydroxycinnamic acids	77.10	80.20	78.09	75.73	75.98	76.45	73.55
Flavonols							
Hyperoside	1.35 ± 0.09 ^bc^	1.60 ± 0.15 ^a^	1.44 ± 0.08 ^bc^	1.28 ± 0.08 ^c^	1.47 ± 0.12 ^ab^	1.36 ± 0.14 ^bc^	1.20 ± 0.12 ^c^
Rutin	4.12 ± 0.20 ^b^	4.47 ± 0.22 ^a^	4.36 ± 0.18 ^a^	4.08 ± 0.12 ^b^	4.23 ± 0.12 ^ab^	4.05 ± 0.10 ^b^	3.78 ± 0.14 ^c^
Isoquercetin	3.28 ± 0.14 ^c^	3.69 ± 0.20 ^a^	3.35 ± 0.12 ^bc^	3.39 ± 0.14 ^bc^	3.55 ± 0.12 ^ab^	3.05 ± 0.10 ^d^	2.89 ± 0.16 ^d^
Isorhamnetin-glycoside	1.96 ± 0.23 ^ab^	2.02 ± 0.12 ^ab^	2.06 ± 0.12 ^a^	1.82 ± 0.08 ^b^	2.05 ± 0.10 ^a^	1.90 ± 0.09 ^ab^	1.63 ± 0.08 ^c^
Σ_Flavonols_	10.71	11.78	11.21	10.57	11.30	11.36	9.50
Anthocyanins							
Cyanidin-glucoside	5.76 ± 0.14 ^a^	5.50 ± 0.12 ^b^	5.15 ± 0.10 ^c^	5.80 ± 0.18 ^a^	5.50 ± 0.12 ^b^	5.32 ± 0.26 ^bc^	4.80 ± 0.12 ^d^
Cyanidin-rutinoside	19.32 ± 0.34 ^b^	19.98 ± 0.32 ^a^	17.59 ± 0.32 ^e^	19.05 ± 0.28 ^b^	18.60 ± 0.30 ^c^	18.03 ± 0.22 ^d^	17.58 ± 0.20 ^e^
Peonidin-glucoside	2.21 ± 0.14 ^a^	1.67 ± 0.12 ^c^	1.74 ± 0.10 ^c^	2.04 ± 0.16 ^ab^	2.05 ± 0.15 ^ab^	2.05 ± 0.12 ^ab^	1.98 ± 0.10 ^b^
Σ _Anthocyanins_	27.29	27.06	24.48	26.89	26.15	25.40	24.36
Σ _Phenolic compounds_	115.10	119.04	113.78	113.19	113.46	113.21	107.41

W0–W6 represent samples collected during fermentation, where W0 is the initial must and W1–W6 correspond to 1–6 days of fermentation, respectively. Different letters indicate statistically significant (*p* < 0.05) differences between the quantitative values of compounds in plum wine at different fermentation times. TPC is expressed as mg gallic acid equivalents (GAE), TFC as mg catechin equivalents (CE), TAC as mg cyanidin-3-glucoside equivalents (CGE), and antioxidant activity as mmol Trolox equivalents (TE). W0: initial must after 3 days of maceration.

## Data Availability

The original contributions presented in the study are included in the article; further inquiries can be directed to the corresponding author.
